# Summary of current evidence for digital health interventions in the screening and management of postpartum depression

**DOI:** 10.3389/fpsyt.2026.1933085

**Published:** 2026-09-16

**Authors:** Tingting Xiang, Shuting Zou, Wei Huang, Manfeng Huang, Ziqi Gao, Ruifang Zhang, Limin Huang

**Affiliations:** 1 First Department of Obstetrics, Hunan Provincial Maternal and Child Health Care Hospital, Changsha, Hunan, China; 2 Department of Postpartum Rehabilitation, Hunan Provincial Maternal and Child Health Care Hospital, Changsha, Hunan, China; 3 Department of Operating Room, Hunan Provincial Maternal and Child Health Care Hospital, Changsha, Hunan, China

**Keywords:** digital health, evidence summary, intervention, mHealth, postpartum depression

## Abstract

**Aim:**

To identify, appraise, summarise, and synthesise current evidence on digital health strategies for postpartum depression (PPD) screening and management, and to develop practice-oriented recommendations to support evidence-informed clinical nursing practice.

**Methods:**

Using the 6S evidence hierarchy to guide the retrieval sequence, we performed a wide literature search of clinical decision-support resources, guideline repositories, professional organisations’ websites, and major Chinese and international databases from their inception to May 1, 2026. Eligible evidence sources included clinical practice guidelines, evidence summaries, and systematic reviews relevant to digital screening, intervention, follow-up, or continuing care for PPD. Two trained reviewers independently screened the literature, assessed the methodological quality of the publications, extracted evidence statements, and synthesised the recommendations given. This evidence summary followed the reporting specifications of the Fudan University Center for Evidence-based Nursing, which are based on the methodological process of the Joanna Briggs Institute (JBI), including problem establishment, literature retrieval, literature screening, literature evaluation, evidence synthesis and grading, and formulation of practical recommendations. The study registration number is ‘ES202610615’.

**Results:**

In total, 16 publications were identified and included in the present study, comprising 2 guidelines, 13 systematic reviews, and 1 evidence summary. From these, 20 key evidence points were extracted and discussed, and 20 evidence statements were generated and then categorised into 5 domains: digital screening and assessment, delivery modalities, intervention components, continuity of care, and implementation issues.

**Conclusion:**

Digital health technologies can extend the reach of PPD screening and management, but their implementation should be carefully adapted to address local service capacity, user needs, referral pathways, privacy requirements, and adherence risks. The 20 evidence statements summarised in this review could help guide the development of standardised, continuous, and patient-centred digital management models for PPD.

## 1 Introduction

Postpartum depression (PPD) is among the most common mental health problems experienced by mothers after childbirth. The reported prevalence of PPD varies widely across populations and settings, with estimates from Chinese studies ranging from approximately 9% to 55% (1, 2). Clinically, patients with PPD may present with a persistent low mood, anxiety, loss of interest, sleep disturbance, irritability, guilt, impaired functioning, and difficulty caring for the infant (3). Severe cases may be associated with a risk of self-harm, impaired mother–infant bonding, disrupted infant development, and a compromised family wellbeing (4). The timely detection and structured management of PPD therefore remain central to perinatal health services.

Current PPD management generally depends on scale-based screening, clinical interviews, psychological intervention, medication when indicated, and follow-up assessment. In routine practice, however, these components are often poorly connected. For instance, screening may be episodic and dependent on staff availability, while interviews and questionnaire scores can be affected by under-reporting, social desirability effects, stigma, and limited privacy (5). After delivery, women may face issues with childcare demands, fatigue, transportation difficulties, cost concerns, and fear of being labelled in negative ways, all of which can delay them seeking professional help and reduce their adherence to treatment or follow-up (6). Fragmentation across obstetric, primary care, community, and mental health services can further weaken the continuity of care they may receive (7).

Digital health technologies offer a practical route to address several of these issues. Such technologies include web-based programs, mobile applications, WeChat mini-programs, text messaging, teleconsultation, remote follow-up, online peer support, and automated symptom monitoring. These can reduce the time needed and geographical barriers to accessing care, increase privacy, and lower service costs. Recent evidence suggests that eHealth interventions can improve depressive, anxiety, and stress symptoms in perinatal populations and may also strengthen their self-efficacy (8). Digital tools also allow for repeated assessment, longitudinal symptom tracking, rapid feedback, and linkage with referral or continuing-care systems.

Despite this clear potential of digital health technologies, their implementation remains uneven. Furthermore, many current digital interventions target a single phase of care, such as screening or psychoeducation, rather than the full pathway from risk identification to referral, intervention, and long-term monitoring. Moreover, the different functions in such systems are often insufficiently integrated in current systems, which can cause user engagement to decline rapidly, while concerns about privacy, safety, and sustainability remain unresolved. Clinicians, therefore, lack a consolidated evidence-based framework for selecting, adapting, and applying digital approaches to PPD. To address this need, the present evidence summary systematically retrieved, appraised, and synthesised current evidence on digital health interventions for PPD, with particular attention paid to digital screening, intervention delivery, content design, continuity of care, and implementation safeguards.

## 2 Methods

This evidence summary was conducted following the Evidence Summary Reporting Standard developed by the Fudan University Center for Evidence-based Nursing, which is itself based on the methodology of the Joanna Briggs Institute (JBI). Accordingly, the study followed a structured process comprising question formulation, hierarchical literature retrieval, literature screening, methodological appraisal of the literature, evidence extraction and grading, and the formulation of practice-oriented recommendations (9). The study registration number was ES202610615.

### 2.1 Problem establishment

The evidence question for this study was developed using the PIPOST framework described by Zhu et al. (10). Here, the population (the first “P” in PIPOST) comprised postpartum women in the general population, women at a high risk of PPD, and women with diagnosed or suspected PPD; the intervention (I) comprised digital screening, digital health interventions, follow-up, and digitally supported management strategies; evidence users/professionals (P) included nurses, physicians, psychological therapists, and digital platform support staff; outcomes (O) included depressive symptom incidence, the depression scale scores, adherence, dropout, referral, and other management-related indicators; settings (S) included hospitals, community care, and home-based care; and finally, the evidence types (T) included clinical practice guidelines, evidence summaries, systematic reviews, and randomised controlled trials when needed to supplement higher-level evidence.

### 2.2 Literature retrieval method

In this review, the 6S evidence hierarchy, an evidence retrieval framework that organises evidence resources according to their level of synthesis, was used to guide the retrieval sequence, prioritising higher-level synthesised evidence before searching lower-level evidence and primary studies. Specifically, the 6S hierarchy was used to guide the order of evidence retrieval rather than to determine the methodological quality or evidence level of the included sources. First, we searched BMJ Best Practice (British Medical Journal Best Practice), UpToDate, the National Institute for Health and Care Excellence (NICE), the World Health Organization (WHO), the Scottish Intercollegiate Guidelines Network (SIGN), the Joanna Briggs Institute (JBI) Evidence-based Healthcare Database, Yimaitong, the American College of Obstetricians and Gynecologists (ACOG), the American Psychological Association (APA), the Registered Nurses’ Association of Ontario (RNAO), the Cochrane Library, PubMed, Web of Science, Embase, the Cumulative Index to Nursing and Allied Health Literature (CINAHL), the Chinese Biomedical Literature Database (CBM), China National Knowledge Infrastructure (CNKI), VIP Databases, and Wanfang Data databases. The reference lists included in the identified eligible studies were also screened to identify additional relevant sources.

The searches combined controlled vocabulary and free-text terms related to PPD, digital health, and PPD management processes. The terms included: postpartum depression, postnatal depression, perinatal depression, maternal depression, digital health, eHealth, mHealth, telemedicine, telehealth, internet-based intervention, online intervention, mobile application, smartphone application, digital intervention, remote monitoring, screening, diagnosis, follow-up, management, and continuity of care. The search covered all records available up to May 1, 2026 and was limited to Chinese- and English-language literature. Because database syntax and controlled vocabulary differ across platforms, the search terms were adapted to the requirements of each database. The PubMed search strategy is presented in **Figure 1** as a representative example.

**Figure 1:**
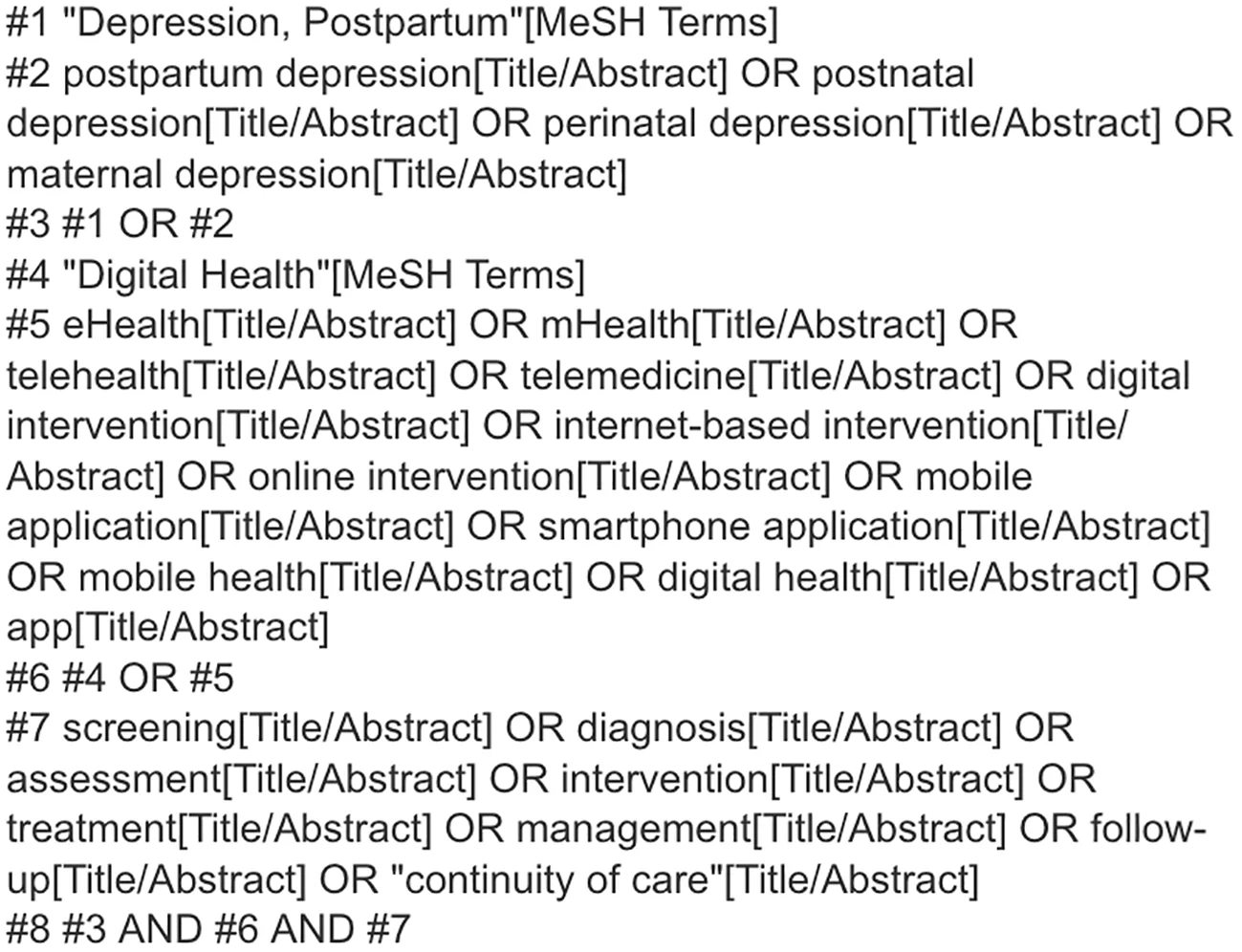
PubMed search strategy for the identification of relevant literature.

### 2.3 Inclusion and exclusion criteria

The literature inclusion criteria were as follows: (1) population: postpartum women, women at an increased risk of PPD, or women with PPD; (2) intervention: digital health interventions, digital screening, digital follow-up, or digitally supported management of PPD; (3) evidence users: healthcare professionals, psychological therapists, or digital platform support staff; (4) outcomes: depressive symptom incidence or severity, screening scores, adherence, dropout, referral, follow-up, or other management outcomes; (5) setting: hospital, community, or home care; and (6) evidence type: guidelines, evidence summaries, systematic reviews, and randomised controlled trials when required. Studies were excluded if they were not published in Chinese or English, if the full text was unavailable, if they were duplicates, or if they were original studies already incorporated into higher-level evidence sources, unless direct appraisal was necessary.

### 2.4 Literature screening

Retrieved records were imported into EndNote and duplicates were removed. Two researchers with training in evidence-based methodology independently screened the titles, abstracts, and keywords. Potentially eligible records were then reviewed by examining their full text. The inclusion and exclusion criteria were discussed by the reviewers before formal screening to ensure their consistent understanding of the eligibility rules. Disagreements about the literature eligibility, classification, or quality appraisal were discussed with a third evidence-based nursing expert until consensus was reached. The literature screening and selcetion process is illustrated in **Figure 2**.

**Figure 2:**
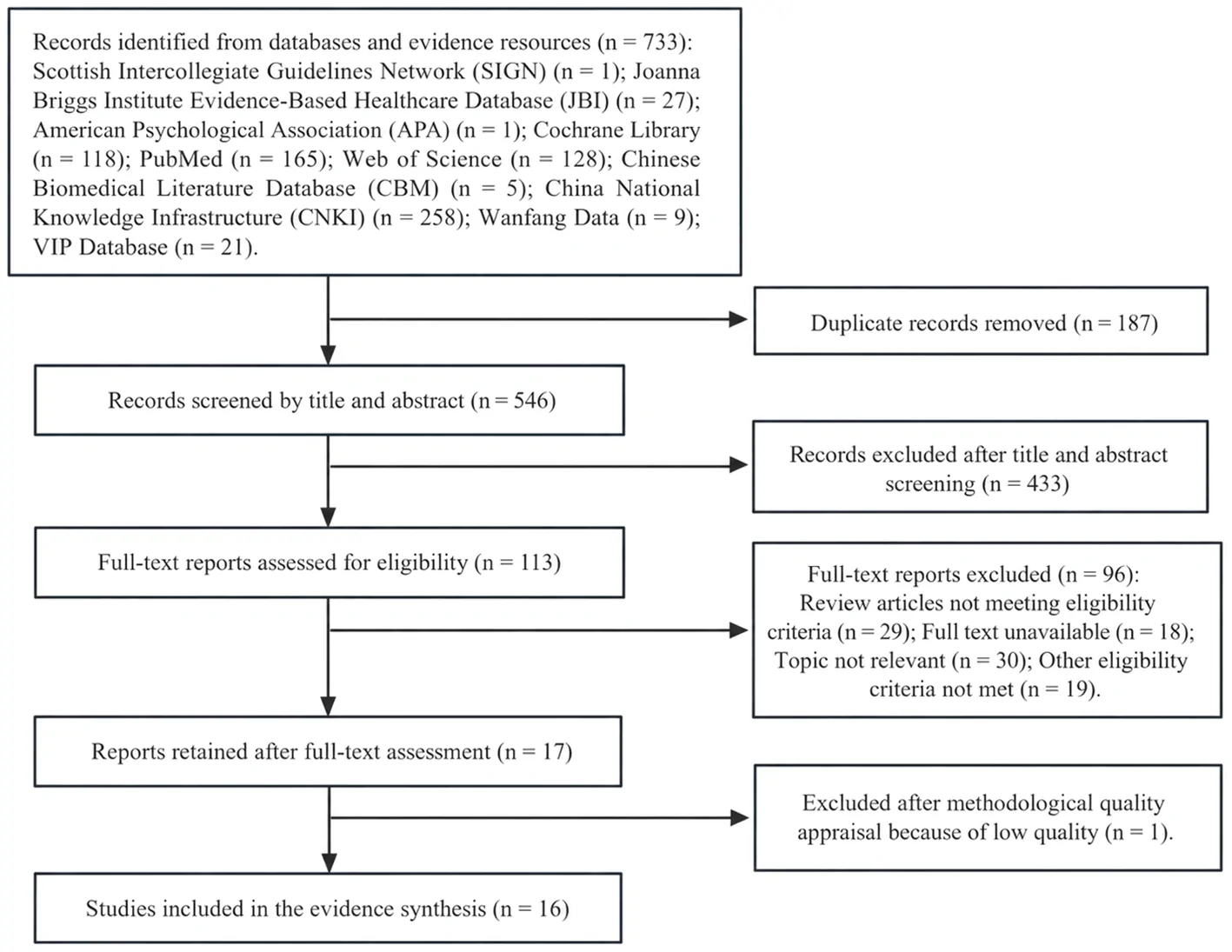
Flow diagram of the literature screening and selection process.

### 2.5 Literature quality evaluation criteria

Clinical practice guidelines were assessed using the Appraisal of Guidelines for Research and Evaluation II (AGREE II) instrument. Systematic reviews were appraised using the JBI critical appraisal checklist for systematic reviews. The methodological basis and reporting standards for these appraisal tools are cited accordingly. For the evidence summaries and other synthesised evidence sources, the references supporting each relevant evidence statement were traced to identify the underlying evidence and study design. The corresponding critical appraisal tool was then selected according to the methodological design of the underlying study. All appraisals were performed by graduate-level nursing researchers who had completed evidence-based practice training. When evidence statements overlapped or conflicted, priority was given to higher-level evidence, evidence from studies with a stronger methodological quality, and more recent publications. The preliminary evidence synthesis was then reviewed and refined through an expert panel consultation process to ensure the clinical applicability and contextual relevance of the final recommendations.

### 2.6 Evidence synthesis and grading

Evidence related to digital PPD screening, intervention, follow-up, and continuing care was extracted from the eligible sources. The extracted information included the author, source, publication year, evidence type, topic, and key findings. Evidence addressing similar clinical or implementation issues was compared and grouped into thematic categories. The key findings were then synthesised into preliminary evidence statements and subsequently reformulated into practice-oriented recommendations specifying the recommended action and, where applicable, the target population or clinical context. The research team reviewed the wording of each recommendation to ensure consistency with the supporting evidence.

When evidence statements overlapped, conceptually similar findings were integrated to avoid duplication. When findings were inconsistent, priority was given to evidence with a higher level, stronger methodological quality, and from more recent publications.

The JBI Evidence Pre-grading and Recommendation Level System (2014 edition) was used to assign the evidence levels and recommendation grades. For the synthesised evidence sources, the supporting original evidence was traced where necessary, and the evidence level was determined according to the underlying study design. When several sources supported the same recommendation, the highest applicable evidence level was retained. Recommendation strength was classified as Grade A or Grade B according to the JBI recommendation criteria, taking into account the balance of benefits and harms, the quality and consistency of the supporting evidence, resource implications, and patient values and experiences (11).

### 2.7 Expert panel consultation

To enhance the clinical applicability and contextual relevance of the synthesised evidence, a panel of 12 experts was convened following the preliminary evidence synthesis. The panellists were purposively recruited from three tertiary hospitals in Hunan Province, China. Eligibility criteria for panel selection included at least 15 years of clinical or research experience in obstetrics, psychiatry, or mental health nursing and a minimum of an intermediate professional title. The final panel comprised 2 medical experts and 10 nursing experts, including 3 with senior, 7 with associate senior, and 2 with intermediate professional titles. All the panellists confirmed they had no conflicts of interest to declare before the consultation.

One week before the structured group meeting, the panellists received a documentation package containing the draft evidence statements, the JBI grading system, and relevant background literature. During the 2-hour meeting, each statement was presented and discussed sequentially under the moderation of an independent chair. The panellists rated each statement on a 5-point Likert scale (1 = strongly disagree to 5 = strongly agree) across three dimensions: clarity, clinical relevance, and feasibility. Consensus was prospectively defined as ≥80% agreement, corresponding to at least 10 of 12 experts rating an item as 4 or 5. Items achieving ≥80% agreement were retained with minor wording adjustments. Items achieving 60%–79.9% agreement (8–9 of 12 experts) were discussed, revised, and re-voted on during the same meeting. Items receiving <60% agreement (≤7 of 12 experts) were considered for merging or exclusion. The mean authority coefficient (Cr) of the panel was 0.95, calculated using the standard formula Cr = (Cs + Ca)/2, where Cs represents familiarity with the topic and Ca represents the basis of judgement, including theoretical analysis, clinical experience, literature reference, and personal intuition. A Cr value ≥ 0.70 is conventionally considered acceptable in Chinese expert consultation studies.

## 3 Results

### 3.1 General information on the included literature

The search identified 733 records of relevance to our study. After excluding duplicate records, followed by title and abstract screening, full-text review, and quality appraisal, 16 publications remained, comprising 2 guidelines (12, 13), 13 systematic reviews (8, 14–25) and 1 JBI best-practice/evidence summary. **Table 1** summarises the basic characteristics of these literature sources.

**Table 1:** Characteristics of the included literature (n=16).

Included literature	Year of publication (year)	Source	Type	Theme
Vigod et al. (12)	2025	PubMed	Guideline	Clinical Practice Guidelines for Perinatal Mood, Anxiety and Related Disorders
SIGN (13)	2023	SIGN	Guideline	Perinatal Mental Health Conditions
Xie et al. (8)	2024	PubMed	Systematic review	Effects of eHealth Interventions on Perinatal Depression
Lewkowitz et al. (14)	2024	AJOG	Systematic review	Effects of Digital Health Interventions on Postpartum Depression or Anxiety
Lau et al. (15)	2024	PubMed	Systematic review	Effects of Digital Health Interventions on Psychological Outcomes in Perinatal Women
Miura et al. (16)	2023	PubMed	Systematic review	App-Based Interventions for the Prevention of Postpartum Depression
Stentzel et al. (17)	2023	PubMed	Systematic review	Effectiveness of Telehealth Interventions on Postpartum Mental Health-Related Outcomes
Spadaro et al. (18)	2022	PubMed	Systematic review	Evaluation of Screening Applications for Perinatal Mental Health
Zhou et al. (19)	2022	Web of Science	Systematic review	Effectiveness of Mobile Health Interventions on Postpartum Depression
Zhao et al. (20)	2021	PubMed	Systematic review	Effectiveness of Telehealth Interventions for Women with Postpartum Depression
Lau et al. (21)	2021	PubMed	Systematic review	Effectiveness of Digital Psychotherapeutic Interventions for Perinatal Women
Gao et al. (22)	2021	Wangfang	Systematic review	Internet-Based Intervention Therapy for Postpartum Depression: A Meta-Analysis of Treatment Effects
Dol et al. (23)	2020	PubMed	Systematic review	Effects of Perinatal Mobile Health Interventions on Maternal Psychosocial Outcomes
Li et al. (24)	2020	CNKI	Systematic review	Effects of Web-Based Cognitive Behavioural Therapy for Postpartum Depression: A Meta-Analysis
Lee et al. (25)	2016	PubMed	Systematic review	Web-Based Interventions for the Prevention and Treatment of Perinatal Mood Disorders
JBI (2020)	2020	JBI	Best practice	Effects of Mobile Health Interventions on Maternal Psychosocial Outcomes During the Perinatal Period

AJOG, American Journal of Obstetrics and Gynecology.

### 3.2 Quality evaluation results of the included literature

#### 3.2.1 Guidelines

Two guidelines were appraised using the AGREE II instrument, namely SIGN (13) Vigod et al. (12), which were rated as Grade A and Grade B, respectively. The standardised scores across the six AGREE II domains, together with the number of domains meeting the ≥60% and ≥30% thresholds used for overall grading, are presented in **Table 2**. Higher standardised scores indicate a better performance in the corresponding domain.

**Table 2:** Quality evaluation results of the included guidelines (n=2).

Included literature	Standardised scores in various domains (%)						≥60%	≥30%	Recommendation level
	Scope and purpose	Stakeholder involvement	Rigour of development	Clarity of presentation	Applicability	Editorial independence			
Vigod et al. (12)	88.89	80.56	60.42	78.93	77.08	80.62	5	0	B
SIGN (13)	88.89	80.56	86.98	97.22	77.08	72.92	6	0	A

#### 3.2.2 Systematic reviews

Thirteen systematic reviews were evaluated using the JBI appraisal checklist for systematic reviews. Most of the reviews met the major methodological criteria; the main weaknesses identified in some of the literature sources were an incomplete reporting of the search strategy themes, an inadequate coverage of the searched resources, and a limited assessment of publication bias (**Table 3**).

**Table 3:** Quality evaluation results of the included systematic reviews (n=13).

Included literature	①	②	③	④	⑤	⑥	⑦	⑧	⑨	⑩	⑪
Xie et al. (8)	Y	Y	Y	Y	Y	Y	Y	Y	Y	Y	Y
Lewkowitz et al. (14)	Y	Y	Y	Y	Y	Y	Y	Y	Y	Y	Y
Lau et al. (15)	Y	Y	Y	Y	Y	N	Y	Y	Y	Y	Y
Miura et al. (16)	Y	Y	Y	Y	Y	Y	Y	Y	Y	Y	Y
Stentzel et al. (17)	Y	Y	Y	Y	Y	Y	Y	Y	N	Y	Y
Spadaro et al. (18)	Y	Y	Y	Y	Y	Y	Y	Y	Y	Y	Y
Zhou et al. (19)	Y	N	Y	Y	Y	Y	Y	Y	Y	N	Y
Zhao et al. (20)	Y	Y	Y	Y	Y	Y	Y	Y	Y	Y	Y
Lau et al. (21)	Y	Y	Y	Y	Y	Y	Y	Y	Y	Y	Y
Gao et al. (22)	Y	Y	N	Y	Y	Y	Y	Y	Y	Y	Y
Dol et al. (23)	Y	Y	N	Y	Y	Y	Y	Y	Y	Y	Y
Li et al. (24)	Y	Y	Y	Y	Y	Y	Y	Y	Y	Y	Y
Lee et al. (25)	Y	Y	Y	N	Y	Y	Y	Y	Y	Y	Y

Y, yes; N, no. Appraisal items: 1, clarity of evidence statements; 2, appropriateness of eligibility criteria; 3, adequacy of the search strategy; 4, sufficiency of databases or resources searched; 5, appropriateness of quality-appraisal criteria; 6, independent appraisal by at least two reviewers; 7, measures to reduce extraction error; 8, appropriateness of synthesis methods; 9, assessment of publication bias; 10, recommendations based on review results; and 11, appropriateness of directions for further research.

#### 3.2.3 Quality evaluation results of the recommended practices in the included literature

The included JBI evidence summary was treated as a synthesised evidence source, and its cited evidence was traced where necessary to support evidence extraction and grading.

### 3.3 Results from the expert panel discussions

Following the preliminary evidence synthesis, an expert panel meeting was convened, during which 12 experts jointly reviewed the drafted evidence statements for clarity, clinical relevance, feasibility, and contextual applicability. Based on the panel feedback, the wording and practical interpretation of the evidence statements were refined. Finally, 20 evidence statements were prepared, classified into five domains: digital screening and assessment, digital intervention modalities, digital intervention content, digital continuity of care, and implementation considerations. Their evidence levels and recommendation grades are presented in **Table 4**.

**Table 4:** Summary of evidence statements and recommendations for digital health interventions in the screening and management of PPD (n = 20).

Category	Recommendations based on the available evidence	Level	Recommendation level
Digital screening and assessment	1. Embed standardised screening tools (e.g. EPDS) into digital platforms for initial identification and dynamic assessment (Lau et al., 2024; Zhou et al., 2022) (15, 19).	1b	A
	2. Combine multidimensional scales (anxiety, stress, insomnia) for comprehensive assessment (Spadaro et al., 2022) (18).	5b	A
	3. Digital screening tools should support result recording and trend tracking to enable longitudinal monitoring of the psychological status (Lewkowitz et al., 2024) (14).	5c	B
	4. Provide clear referral pathways or professional support for screen-positive individuals to achieve tiered management (SIGN et al., 2023) (13).	5b	A
Digital intervention modalities	5. Prioritise mobile applications (apps) as the primary intervention delivery tool (Xie et al., 2024; Miura et al., 2023) (8, 16).	1a	A
	6. Use SMS, telephone calls, or online communities as supplementary support (Lau et al., 2024; Zhou et al., 2022) (15).	1c	B
	7. Use video or audio formats to enhance the comprehensibility of intervention content (Xie et al., 2024; Gao et al., 2021) (8, 22).	5c	B
	8. Combine self-guided interventions with remote professional guidance to improve effectiveness (Zhao et al., 2021) (20).	1a	A
Digital intervention content	9. Use cognitive behavioural therapy (CBT) as the core intervention, including cognitive restructuring, behavioural activation, etc. (Xie et al., 2024; Stentzel et al., 2023; Li et al., 2020) (8, 17, 24).	1a	A
	10. Adopt a modular design (e.g. staged courses plus homework exercises) to improve structure and adherence (Stentzel et al., 2023) (17).	5c	B
	11. Include psychoeducation modules to provide knowledge of the disease, parenting information, and self-management skills (Lau et al., 2024; Lau et al., 2021) (15, 21).	5b	A
	12. Include peer support modules (e.g. group chats or communities) to enhance social support and experience-sharing (Dol et al., 2020) (23).	1c	B
	13. Incorporate mindfulness training to improve emotion regulation (Lau et al., 2021) (21).	1a	B
	14. Provide mood recording or tracking functions to facilitate self-awareness and regulation (Stentzel et al., 2023) (17).	5c	B
Digital continuity of care	15. Integrate screening, intervention, and follow-up functions on a digital platform to achieve a continuous care pathway (Vigod et al., 2024) (12).	5b	A
	16. Support the sharing of screening results with healthcare systems to facilitate clinical decision-making and tiered referral (Spadaro et al., 2022) (18).	5c	B
	17. Use online follow-up or automated reminders to promote continuity and adherence (Dol et al., 2020) (23).	1b	A
Directions for optimisation	18. Address low adherence in digital interventions by enhancing interactive design and personalised feedback to increase user engagement (Lee et al., 2016) (25).	1b	A
	19. Strengthen data security and privacy protection mechanisms (Spadaro et al., 2022) (18).	5b	A
	20. Select appropriate technology pathways based on target population characteristics for intervention delivery (Dol et al., 2020) (23).	5c	A

For detailed implementation examples for each recommendation, see **Supplementary Table S1**.

## 4 Discussion

### 4.1 Digital screening and assessment

Timely identification is the first step in reducing the clinical and developmental consequences of postpartum depression (PPD), as delayed recognition may postpone psychological support, referral, and risk management for both mothers and infants (26). This is covered in Recommendations 1 and 2 in **Table 4**, which support the incorporation of standardised screening instruments into digital platforms for both the initial detection and repeated assessment of PPD. Among the available tools, the Edinburgh Postnatal Depression Scale (EPDS) remains the most widely used instrument, but digital systems also allow depression screening to be combined with assessments of anxiety, perceived stress, sleep disturbance, and other related symptoms. This broader and repeated assessment structure can change screening from an isolated encounter into a longitudinal monitoring process, making it easier to detect symptom fluctuation and emerging risk over time. Consistent with this interpretation, previous evaluations of perinatal mental health applications have shown that many platforms already include the EPDS or a similar scale, together with functions for self-assessment and result recording (18). The perceived acceptability of digital self-assessment also appears favourable, with evidence suggesting that user responses are not substantially affected by whether screening is completed on paper or electronically (27). These findings indicate that digital screening is feasible for clinical and community-based use, provided that the tools are embedded within a broader care pathway rather than used as stand-alone questionnaires.

Recommendation 4 in **Table 4** further suggests that positive screening results should trigger clear referral pathways or professional support. Indeed, screening has limited clinical value if it only identifies risk without directing women to the appropriate support. Therefore, digital screening systems should include explicit referral pathways, professional consultation options, emergency contact information, and mechanisms for stepped or tiered management. Current guidelines similarly emphasise the importance of coordination among maternity services, primary care, and mental health professionals as a prerequisite for effective perinatal mental health care (14). However, existing applications do not always provide sufficient guidance after a positive screening result, and some lack clear instructions for urgent referral or professional follow-up when high-risk symptoms are detected (18). This gap suggests that future digital screening tools should be evaluated not only by their usability or screening accuracy, but also by their ability to connect assessment results with subsequent intervention and referral.

Emerging technologies may further extend the scope of digital screening. In particular, machine-learning models and wearable sensors have already been explored for early risk prediction using behavioural, physiological, or neurophysiological signals (28, 29). For example, wrist-worn electrodermal activity and other sensor-derived indicators have been combined with deep-learning approaches to classify the severity of depressive symptoms (30). These approaches are promising because they may capture dynamic changes that cannot be fully detected through self-reporting scales alone. Nevertheless, most predictive models remain at the stage of algorithm development or preliminary validation, and their generalisability, interpretability, data security, and integration into routine maternal care still require further evaluation. Thus, the immediate priority is not to replace standardised scale-based screening, but rather to build clinically actionable digital screening systems that combine validated questionnaires, longitudinal symptom tracking, referral decision support, and, where appropriate, cautiously validated predictive technologies.

### 4.2 Digital intervention modalities

Digital health technologies have expanded the delivery options for postpartum depression (PPD) care by reducing the barriers related to the time needed to access services, the distance that may be involved, childcare responsibilities, and the limited access to face-to-face psychological services for many patients. The evidence summarised in this review indicates that mobile applications are increasingly being used as a primary delivery channel, largely because they are portable, scalable, and compatible with repeated symptom monitoring and self-management. Compared with web-based platforms, app-based interventions may offer greater convenience and promote more frequent user contact, particularly when they include reminders, interactive modules, and personalised feedback (31). However, mobile applications should not be interpreted as universally superior to other delivery formats. Their effectiveness depends on the intervention content, their usability, user engagement, the digital literacy of users, and the availability of professional support. Therefore, apps should be best understood as one important platform within a broader digital intervention system rather than as a stand-alone solution.

SMS messages, telephone calls, web platforms, video consultations, and online communities can all serve as complementary modalities, especially for women with different levels of digital access or support needs (14). Each offers its own benefits. For instance, SMS-based support is simple, low-cost, and accessible, and may be useful for reminders, brief psychoeducation, and follow-up prompts. Telephone follow-up can provide direct interpersonal contact and may be particularly valuable for women who require reassurance, symptom clarification, or referral guidance. Online communities and peer-support groups may strengthen perceived social support, although they require appropriate moderation to avoid misinformation, privacy risks, or emotional contagion. Video and audio materials can also improve the comprehensibility of intervention content by converting written psychological education into more accessible and engaging formats. These features suggest that the choice of modality should be matched to the target population, intervention intensity, available clinical resources, and local implementation context.

The evidence also supports the value of blended intervention models that combine self-guided digital modules with remote professional input. However, while purely self-directed interventions may increase accessibility, they often face problems of low adherence, limited personalisation, and a delayed response to worsening symptoms. In contrast, interventions supported by clinicians, nurses, counsellors, or trained facilitators can provide feedback, reinforce engagement, and connect women with higher-intensity care when needed. For example, web-based cognitive behavioural therapy combined with telephone support has been reported to improve depression, anxiety, and stress symptoms in women with postnatal depression, highlighting the added value of human support in digital psychological care (32). Similarly, WeChat mini-programs and specialised remote management applications developed in Chinese clinical settings suggest that online–offline integration may be feasible when digital tools are embedded within structured professional follow-up and family-based support (33). Overall, the optimal strategy is not to rely on a single technological format, but to construct a flexible, multi-channel model that balances accessibility, clinical safety, engagement, and continuity of care.

### 4.3 Digital intervention content

Recommendations 9–14 in **Table 4** cover possible digital interventions for postpartum depression (PPD) and suggest that they should include both structured psychological components and supportive self-management functions. Cognitive behavioural therapy (CBT), particularly cognitive restructuring and behavioural activation, remains the most frequently used and best-supported therapeutic framework in digital PPD interventions. Internet-delivered CBT can help women identify maladaptive thoughts, increase adaptive behaviours, and develop coping strategies for mood disturbance during the perinatal period (17). However, CBT should not be presented merely as a digitised version of face-to-face psychotherapy. In a digital context, its effectiveness depends on how well therapeutic content is translated into brief, understandable, interactive, and developmentally appropriate modules that fit the daily routines of postpartum women.

Beyond CBT-based content, psychoeducation, mindfulness training, peer support, and mood tracking represent important complementary components. In particular, psychoeducation can improve women’s understanding of PPD, normalise help-seeking, and provide practical information on parenting, sleep, emotional regulation, and self-care (34). Mindfulness-based exercises may help reduce emotional reactivity and improve coping with stress, although their effects are likely to depend on the intervention intensity, adherence, and user preference. Peer-support functions, such as moderated online groups or discussion communities, may reduce social isolation and provide experiential support; however, these functions require careful supervision to protect privacy, prevent misinformation, and avoid the spread of distressing content. Mood recording and symptom-tracking tools can enhance self-awareness and provide clinicians with longitudinal information, but their value depends on whether recorded data are linked to feedback, risk alerts, or follow-up actions.

Therefore, the key issue is not simply to increase the number of intervention modules, but to define the active ingredients of digital intervention and to organise them into a clinically coherent pathway. A modular design may allow different components to be matched to the symptom severity, risk profile, digital literacy, and individual preference. This is consistent with stepped-care principles, in which low-intensity self-management tools may be offered to women with mild symptoms, while women with persistent, worsening, or high-risk symptoms should receive professional support, referral, or more intensive psychological intervention (35). Artificial intelligence may eventually support this process by helping to stratify risk, recommend modules, or personalise feedback. Nevertheless, AI-based tailoring should be introduced cautiously, with attention to algorithm validation, transparency, privacy protection, and clinical accountability. Furthermore, it is considered that future digital PPD interventions should move from broad content aggregation toward mechanism-informed, user-centred, and clinically actionable intervention designs.

### 4.4 Digital continuity of care

A major advantage of digital health lies in its potential to link screening, assessment, intervention, referral, and follow-up within a continuous care pathway. Rather than functioning as isolated tools, digital platforms should support closed-loop management by recording users’ mental health status, delivering tailored interventions, prompting follow-up, and facilitating referral when risk is identified. However, current digital tools for perinatal mental health remain largely fragmented. A scoping review reported that only a small proportion of mobile health tools covered the full period from pregnancy to postpartum, and many platforms focussed on prevention, screening, or treatment as separate functions rather than as integrated services (36). Similarly, while Shenzhen developed a PPD screening and intervention platform that incorporated user, provider, and management interfaces for screening, referral, intervention, and case tracking, subsequent intervention participation remained below 20%, indicating that identification alone may not ensure effective engagement or the continuity of care for PPD patients (37).

Data sharing and system interoperability are therefore central to the digital continuity of care. Screening results should be transferable to healthcare systems to support clinical decision-making, tiered referral, and coordinated follow-up. The Maternal–Infant Integrated Digital Mental Health Service project provided a practical example of a model that combines monitoring, risk alerts, digital intervention, decision support, provider training, and follow-up guidance (38). Nevertheless, such models require clear workflow design, defined responsibilities across care settings, secure data governance, and adequate training for healthcare providers. Future implementation should, therefore, prioritise not only platform construction, but also cross-system linkage and standardised care pathways that ensure women identified as at-risk receive timely and appropriate support.

### 4.5 Implications and limitations for clinical practice

Digital health interventions can improve access to PPD management by reducing the barriers related to accessing care, such as time, geography, cost, transportation, and childcare responsibilities. They may also be acceptable to many postpartum women, as shown by the reported favourable user satisfaction in studies of mobile mental health interventions (39). In clinical practice, digital tools may therefore serve as useful complements to routine maternal and mental health services, particularly for screening, psychoeducation, symptom tracking, follow-up reminders, and low-intensity psychological support.

However, several implementation challenges remain and should be addressed before the wider dissemination of such digital tools and services. First, adherence remains a major limitation. Attrition in digital interventions varies widely across perinatal studies, suggesting that engagement is strongly influenced by the content quality, usability, personal relevance, and the degree of professional support (40, 41). Second, safety and privacy require careful regulation. Some applications lack clear risk alerts, referral instructions, or emergency support information, which may be problematic for women with severe depressive symptoms or a self-harm risk (42). Third, the intervention design should account for the digital divide. Differences in device access, internet availability, and digital literacy may affect equity and effectiveness (43). For women with limited digital literacy, simple apps, SMS, telephone follow-up, and offline guidance may be more appropriate; for women with higher digital access, video-based modules, online communities, and interactive self-management tools may be added. Overall, digital interventions should not replace professional care, but should be integrated into safe, standardised, and context-sensitive service pathways.

## 5 Conclusion

This study identified and synthesised 20 evidence statements from the current literature on digital health interventions for the screening and management of postpartum depression. The evidence supports an emerging care model that integrates digital screening, structured intervention content, multi-channel delivery, continuity of care, and implementation safeguards. In practice, this evidence should be carefully adapted according to local clinical resources, patient preferences, digital literacy, privacy requirements, and the available referral pathways. Future research should focus on improving adherence, validating integrated care models, and developing safe, standardised, and scalable digital management strategies for postpartum depression.

## Data Availability

The raw data supporting the conclusions of this article will be made available by the authors, without undue reservation.
