# Supplementary material for: Summary of current evidence for digital health interventions in the screening and management of postpartum depression
Source: Front Psychiatry. 2026 Sep 16;17:1933085. doi: 10.3389/fpsyt.2026.1933085 (PMC13624077; doi:10.3389/fpsyt.2026.1933085)
Supplement: Supplementary file 1 [file Table1.docx]

| **Supplementary Table S1.** Implementation examples for each recommendation statement | | |
| --- | --- | --- |
| Category | Recommendations | Implementation example |
| Digital screening and assessment | 1. Embed standardised screening tools (e.g. EPDS) into digital platforms for initial identification and dynamic assessment. | Integrate the Edinburgh Postnatal Depression Scale (EPDS) into a hospital WeChat mini-program. Postpartum women complete the scale via their mobile devices during routine postnatal visits. The system automatically scores the responses and, if the total score exceeds the predefined cut-off (e.g. ≥13), it generates an alert to the responsible clinician and prompts a follow-up assessment or referral. |
|  | 2. Combine multidimensional scales (anxiety, stress, insomnia) for comprehensive assessment. | In addition to the EPDS, include the Generalised Anxiety Disorder-7 (GAD-7) for anxiety, the Perceived Stress Scale (PSS) for stress, and the Insomnia Severity Index (ISI) for sleep disturbance within the same digital platform. The system displays a composite risk profile to clinicians, enabling a more holistic understanding of the woman's mental health status beyond depressive symptoms alone. |
|  | 3. Digital screening tools should support result recording and trend tracking to enable longitudinal monitoring of the psychological status. | The digital platform stores all historical screening results for each user, allowing clinicians and the women themselves to visualise symptom trajectories over time (e.g. via line charts). This facilitates the early detection of deterioration and enables data-driven adjustments to treatment plans. |
|  | 4. Provide clear referral pathways or professional support for screen‑positive individuals to achieve tiered management. | When a woman screens positive, the platform automatically generates a standardised referral message containing the screening results, basic demographic information, and contact details, and sends it to the designated mental health professional or community health centre. The system also provides a list of available referral options (e.g. online psychological counselling, face-to-face psychiatric consultation, or community-based peer support) stratified by symptom severity. |
| Digital intervention modalities | 5. Prioritise mobile applications (apps) as the primary intervention delivery tool. | Develop a standalone mobile application specifically designed for postpartum mental health, available for download via official app stores. The app serves as the central hub for delivering all intervention components, including psychoeducation, cognitive behavioural therapy (CBT) modules, and mood tracking, ensuring that women can access support anytime and anywhere. |
|  | 6. Use SMS, telephone calls, or online communities as supplementary support. | In addition to the core app-based intervention, send automated SMS reminders to users three times a week to complete their daily mood check-in. Offer optional telephone coaching sessions once every two weeks for women who prefer verbal communication. Create a moderated online community within the app where users can share experiences and provide mutual support. |
|  | 7. Use video or audio formats to enhance the comprehensibility of intervention content. | Convert key psychoeducation and CBT module content into short animated videos (5–10 minutes each) and audio recordings. These are embedded within the app alongside text-based materials, allowing users with low health literacy or limited time to engage with the content through their preferred medium. |
|  | 8. Combine self‑guided interventions with remote professional guidance to improve effectiveness. | The app provides self-guided modules that users can complete at their own pace, supplemented by asynchronous professional guidance (e.g. a therapist reviews user responses to homework exercises and provides personalised written feedback via the app within 48 hours). A live one-on-one text or video chat session with a therapist can also be scheduled on demand. |
| Digital intervention content | 9. Use cognitive behavioural therapy (CBT) as the core intervention, including cognitive restructuring, behavioural activation, etc. | The app's core intervention curriculum is structured around evidence-based CBT principles. Modules include: (1) identifying and challenging negative automatic thoughts (cognitive restructuring), (2) scheduling and engaging in pleasurable activities (behavioural activation), and (3) problem-solving skills training. Each module includes interactive exercises and real-life application tasks. |
|  | 10. Adopt a modular design (e.g. staged courses plus homework exercises) to improve structure and adherence. | Structure the app content into sequential stages (e.g. Module 1: Understanding PPD, Module 2: Cognitive Restructuring, Module 3: Behavioural Activation). Each stage includes a core lesson, supplementary reading materials, and a homework assignment that the user completes before progressing to the next stage. Progress is tracked and displayed to motivate continued engagement. |
|  | 11. Include psychoeducation modules to provide knowledge of the disease, parenting information, and self‑management skills. | Develop a comprehensive psychoeducation section within the app covering: (1) what PPD is and its common symptoms, (2) practical parenting tips (e.g. infant sleep training, breastfeeding support), (3) self-management strategies (e.g. stress reduction techniques, time management, and when to seek professional help). The content is presented in a mix of text, infographics, and short videos. |
|  | 12. Include peer support modules (e.g. group chats or communities) to enhance social support and experience-sharing. | Enable a moderated group chat function within the app where users can join small groups (8–10 members per group) facilitated by a trained peer supporter. Groups meet virtually once a week for structured discussions on topics such as "coping with sleep deprivation" or "managing relationship changes after childbirth". An open forum is also available for general questions and experience-sharing. |
|  | 13. Incorporate mindfulness training to improve emotion regulation. | Include a dedicated mindfulness module in the app featuring guided mindfulness meditation exercises (e.g. body scan, breathing exercises, mindful observation) in audio format, ranging from 5 to 20 minutes. Users are encouraged to practice daily, and the app tracks their practice frequency and sends reminders. |
|  | 14. Provide mood recording or tracking functions to facilitate self‑awareness and regulation. | Include a daily mood diary feature where users can rate their mood on a 10-point scale and optionally add free-text notes describing their emotional state, triggers, and coping strategies. The app displays trends over time (e.g. weekly and monthly summaries) to help users identify patterns and triggers, and to facilitate self-monitoring. |
| Digital continuity of care | 15. Integrate screening, intervention, and follow‑up functions on a digital platform to achieve a continuous care pathway. | The platform serves as a one-stop solution: initial screening → risk stratification → personalised intervention delivery → regular follow-up reminders → outcome re-assessment. All data are stored within the same user profile, enabling seamless transitions between different phases of care. For example, when a user completes the intervention phase, the system automatically schedules follow-up assessments at 1, 3, and 6 months post-intervention. |
|  | 16. Support the sharing of screening results with healthcare systems to facilitate clinical decision‑making and tiered referral. | The digital platform is interfaced with the hospital's electronic health record (EHR) system, allowing screening results and intervention progress to be automatically pushed to the woman's primary care provider. Clinicians can access this information during consultations to inform clinical decision-making and to validate the appropriateness of the tiered referral pathway (e.g. primary care management vs specialist referral). |
|  | 17. Use online follow‑up or automated reminders to promote continuity and adherence. | Automatically send follow-up assessment questionnaires (e.g. EPDS) to users via the app at 4, 8, and 12 weeks post-intervention initiation. Send reminder notifications to users who miss scheduled assessments or who have been inactive for more than 7 days. If a user is lost to follow-up, a text message reminder and/or telephone call is triggered to re-engage them. |
| Directions for optimisation | 18. Address low adherence in digital interventions by enhancing interactive design and personalised feedback to increase user engagement. | Incorporate gamification elements such as progress badges, daily streaks, and achievement milestones to increase motivation. Provide personalised feedback based on user activity data (e.g. "You've completed 5 CBT exercises this week—great progress! Continue with Module 3"). Send personalised congratulatory messages when users complete milestones. |
|  | 19. Strengthen data security and privacy protection mechanisms. | Ensure that all user data are encrypted during transmission (using SSL/TLS protocols) and at rest (using AES-256 encryption). Implement strict access controls, allowing only authorised healthcare providers to access user data on a need-to-know basis. |
|  | 20. Select appropriate technology pathways based on the target population characteristics for intervention delivery. | For populations with low digital literacy or limited smartphone access, consider delivering the intervention via SMS-based messaging or interactive voice response (IVR) calls rather than a full-featured mobile app. For populations with high smartphone penetration, a comprehensive app-based intervention with rich media content is more appropriate. Conduct a pre-implementation needs assessment to understand the target population's technology access and preferences before selecting the delivery pathway. |
